# The Relationship of Acupuncture Use to the Endometriosis Risk in Females With Rheumatoid Arthritis: Real-World Evidence From Population-Based Health Claims

**DOI:** 10.3389/fmed.2020.601606

**Published:** 2021-02-22

**Authors:** Wei-Jen Chen, Hanoch Livneh, Chien-Hui Hsu, Ying-To Hu, Ning-Sheng Lai, How-Ran Guo, Tzung-Yi Tsai

**Affiliations:** ^1^Department of Chinese Medicine, Dalin Tzu Chi Hospital, The Buddhist Tzu Chi Medical Foundation, Chiayi, Taiwan; ^2^School of Post-Baccalaureate Chinese Medicine, Tzu Chi University, Hualien, Taiwan; ^3^Graduate Institute of Sports Science, National Taiwan Sport University, Taoyuan City, Taiwan; ^4^Rehabilitation Counseling Program, Portland State University, Portland, OR, United States; ^5^Department of Rehabilitation, Dalin Tzu Chi Hospital, The Buddhist Tzu Chi Medical Foundation, Chiayi, Taiwan; ^6^Division of Allergy, Immunology and Rheumatology, Dalin Tzu Chi Hospital, The Buddhist Tzu Chi Medical Foundation, Chiayi, Taiwan; ^7^School of Medicine, Tzu Chi University, Hualien, Taiwan; ^8^Department of Environmental and Occupational Health, College of Medicine, National Cheng Kung University, Tainan, Taiwan; ^9^Department of Occupational and Environmental Medicine, National Cheng Kung University Hospital, Tainan, Taiwan; ^10^Occupational Safety, Health, and Medicine Research Center, National Cheng Kung University, Tainan, Taiwan; ^11^Department of Nursing, Tzu Chi University of Science and Technology, Hualien, Taiwan; ^12^Department of Medical Research, Dalin Tzu Chi Hospital, The Buddhist Tzu Chi Medical Foundation, Chiayi, Taiwan

**Keywords:** acupuncture, endometriosis, female, rheumatoid arthritis, risk

## Abstract

**Objectives:** Women affected by rheumatoid arthritis (RA) have a higher risk of endometriosis, an estrogen-dependent, chronic inflammatory disease. Though acupuncture has long been a safe and effective therapy for treating inflammatory conditions, it is unclear whether it could prevent the onset of endometriosis. This study aims to determine the effect of acupuncture on the subsequent risk of endometriosis in female RA patients.

**Methods:** Between 1998 and 2010, female subjects with RA were recruited from a nationwide database (5,736 patients; age ≥20 years). Enrolled patients included 2,407 acupuncture users and 2,407 nonusers randomly selected using propensity scores. The occurrence of endometriosis was recorded through the end of 2012. Cox proportional hazards regression was used to estimate the adjusted hazard ratio (HR) associated with acupuncture use.

**Results:** During the follow-up period, 35 acupuncture users and 94 non-users developed endometriosis, with incidence rates of 2.36 and 4.91 per 1,000 person-years, respectively. Acupuncture use was associated with a 55% lower endometriosis risk (adjusted HR, 0.45; 95% confidence interval, 0.31–0.65). Those who received high intensity acupuncture (≥15 packages) had the greatest benefit.

**Conclusions:** Findings suggest that adding acupuncture to conventional therapy may decrease the subsequent endometriosis risk in female RA patients. Prospective randomized trials are recommended to further clarify whether the association revealed in this study supports a causal link.

## Keypoints

- Acupuncture has drawn more attention due to its reliable therapeutic efficacy for the subjects with the inflammatory disease, especially RA.
- To the best of our knowledge, no large population-based studies on the association of acupuncture use with the endometriosis risk in female RA patients have been published.
- The results of multivariable analysis demonstrated that the use of acupuncture was related to a significantly reduced risk of endometriosis, with an adjusted HR (95% CI) of 0.45 (0.30–0.65).
-In the subgroup analysis, the medium- to high-level intensity acupuncture use was found to possibly lessen the risk of having endometriosis for more than 70%.
- We discovered that the post-RA acupuncture use would significantly reduce the risk of endometriosis in a dose-dependent manner.

## Introduction

Rheumatoid arthritis (RA) is a long-term autoimmune disorder that primarily affects the joints. Features of RA are demonstrated in the swelling, tenderness, and damage of synovial joints, which might cause the affected patients ultimately develop progressive functional limitations and physical disability. Of previously employed RA sufferers, about two-thirds experience reduced work capacity after RA onset (1), creating an enormous burden on these patients and on the healthcare system. In the United States, the aggregate incremental expenditures attributed to RA increased from $64.8 billion USD in 1997 to $80.8 billion USD in 2003, an increase of 25% (2). On average, the estimated healthcare cost of RA was approximately US$ 20,919 per affected person, ~3 times higher than for the non-RA patients (3).

Like its chronic inflammation among the patients affected by RA, it may be a driver of numerous chronic illnesses. For example, the systemic inflammation associated with RA has been found to provoke the risk of developing an estrogen-dependent inflammatory disorder, particularly endometriosis. A cohort study using data from the Nurses' Health Study of 114,453 female nurses followed over 22 years showed that those with RA had a 40% higher risk of developing endometriosis than did the general population (4). Endometriosis is a chronic gynecological disorder characterized by the presence of endometrial tissue external to the uterine cavity (5). This illness can cause pelvic inflammation, adhesions, chronic pain, and even infertility or miscarriage (5–7). Notably, most women develop RA between the ages of 30 and 50, the childbearing years. The aggregate conditions associated with endometriosis severely diminish their quality of life and family functioning, even resulting in divorce. Thus, reducing the incidence of endometriosis among RA patients is of high priority.

As a mainstream non-pharmaceutical therapy, acupuncture is commonly regarded as a complementary therapy for patients with chronic diseases (8, 9). Acupuncture has been shown to reduce the expression of inflammatory mediators, modulate the autonomic nervous system, and produce analgesic effects in RA patients (10). The beneficial effect of acupuncture on rheumatic disorders suggests mediation of the body's inflammatory responses through the hypothalamus–pituitary–adrenal axis and autonomic nervous system (11, 12). Thus, acupuncture should be considered when instituting effective treatments for RA patients. Nevertheless, whether acupuncture decreases the risk of endometriosis among RA patients is presently unknown. This study uses a nationwide population-based database to assess the risk of endometriosis among female RA patients treated with or without acupuncture, which is beneficial to provide real-world evidence and improve clinical and policy decisions.

## Methods

### Data Source

The subjects of this cohort study were retrieved from a national representative sample in the Longitudinal Health Insurance Database (LHID), which is managed by the National Health Insurance Administration. The National Health Insurance (NHI) program was instituted in 1995 and now covers over 99% of the citizens in Taiwan. The study cohort used in this study comprises a subset of the NHI program including 1 million randomly sampled persons registered in 2000. The database includes all medical records of these individuals from 1997 to 2012, NHI enrollment files, claims data, and a prescription drug registry. Because randomized stratified systematic sampling methods were used, there were no statistically significant differences between these 1 million insured individuals and the general population (13). Since the LHID consists of de-identified secondary data released to the public for research purposes, the study was exempt from full review by the International Review Board after consulting with the Director of the IRB of our institution (No. B10004021-3).

### Study Population

**Figure 1:**
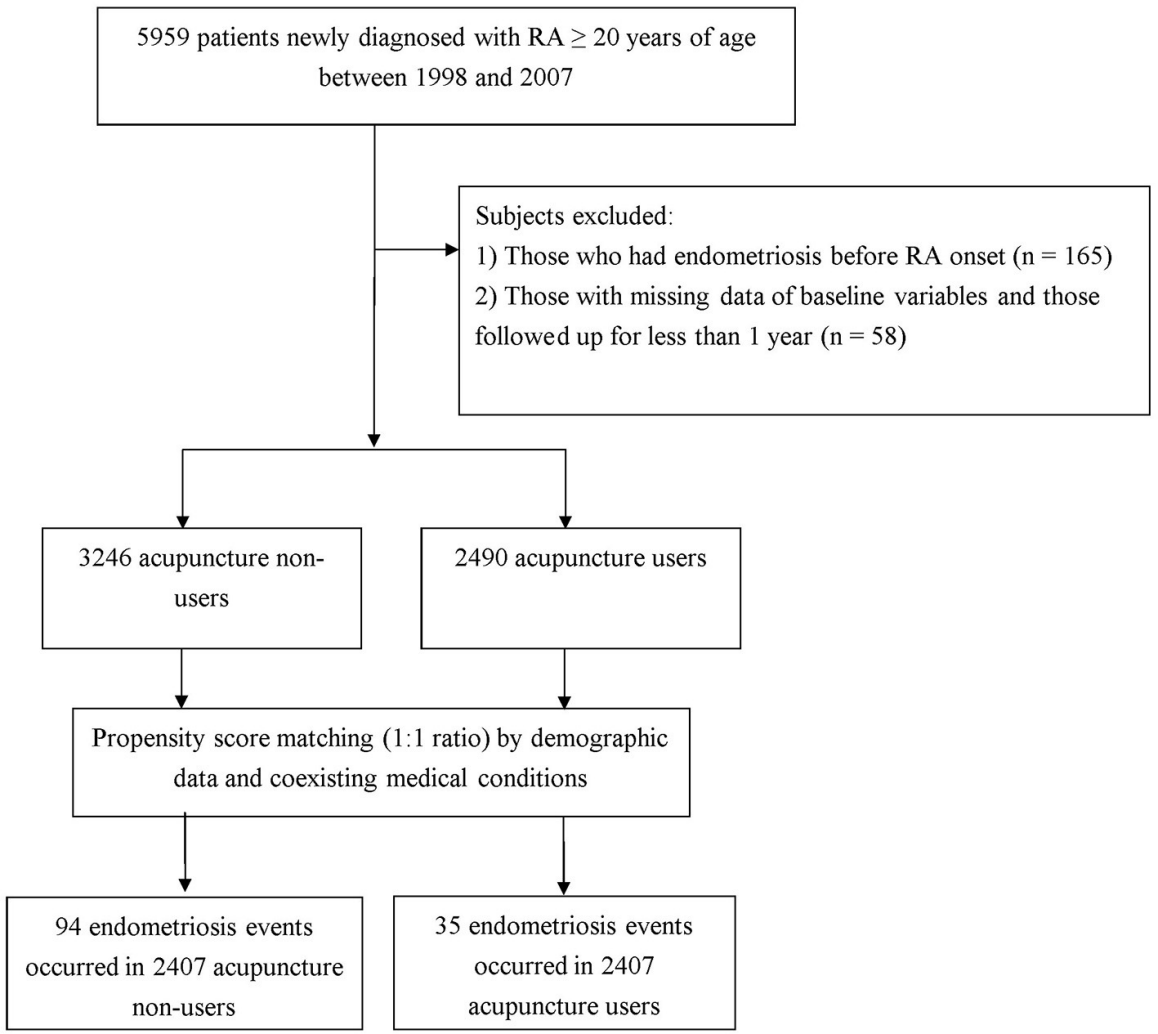
Flow chart of subject inclusion.

The process used to identify and select subjects for this population-based retrospective cohort study is shown in Figure 1. All diagnosed diseases were coded according to the International Classification of Disease, 9th Revision, Clinical Modification (ICD-9-CM). Inclusion criteria were comprised of female sex, age 20 years or older, and being newly diagnosed with RA (ICD-9-CM code 714.0) between 1998 and 2010. To reduce the potential for disease misclassification, only those patients with catastrophic illness certification for RA were recruited. In Taiwan, such certification exempts patients with major illnesses from co-payments. The index date in this study is the date on which the RA patient was approved for catastrophic illness registration. Excluded from this study were 189 subjects diagnosed with endometriosis prior to the date of the first RA diagnosis. In Taiwan, the diagnosis of endometriosis is not made solely on the basis of clinical signs and symptoms, and some examinations, such as physical examination (including pelvic examination), ultrasound, laparoscopy, or magnetic resonance imaging, are required according to the generally accepted diagnostic criteria (14). However, such a diagnosis may be listed on the insurance claim of the first, and even the second, outpatient visit in order to get the examinations reimbursed. To ensure the accuracy and avoid overestimation of the incidence of endometriosis, the present study excluded females who had at least three outpatient service claims or one hospitalization claim with the diagnosis of endometriosis (ICD-9-CM code of 617), dating from 1996 (when computerized claims data from the LHID became available) until the date of the cohort study. Thereafter, we also excluded individuals with missing data on age or sex, as well as those who were not followed fully for 1 year following RA onset (*n* = 34). A final cohort of 5,736 new-onset female RA subjects were candidates for data analysis.

In Taiwan, only certified Chinese medicine physicians can provide acupuncture treatment. According to the program's protocol, six consecutive acupuncture treatments delivered to a patient within 1 month was considered a package of acupuncture (9). Patients receiving at least one package of acupuncture were identified as “acupuncture users,” whereas the remainder were regarded as “acupuncture non-users.” In accordance with this designation, 2,490 patients were classified as acupuncture users. A comparison cohort was randomly selected from the remaining enrollees who did not receive acupuncture. For each patient receiving acupuncture, a patient who did not receive acupuncture was selected, using 1:1 propensity score matching. The propensity score was calculated using logistic regression derived from patients' demographics and baseline comorbidities at enrollment. Ultimately, equal numbers of subjects were enrolled for the two groups (Figure 1). The index date of the follow-up period for patients classified as acupuncture non-users was the date of the first RA diagnosis, whereas that for acupuncture users was the first date of the initiation of acupuncture treatment. All participants were followed from the index date to the date of endometriosis diagnosis, death, withdrawal from the insurance program, or the end of 2012.

### Covariate Assessment

Covariates included demographic characteristics and baseline comorbidities. Demographic characteristics included age, income (estimated by using insurance premium), and the urbanization level of the subject's location of residence. The incomes were stratified into 3 levels in accordance with the income-related insured premium per month: ≤ New Taiwan Dollar (NTD) 17,880; NTD 17,881-NTD 43,900; and ≥ NTD 43,901. Urbanization level was separated into 7 distinct subcategories based on several parameters, which included the population density, percentage of residents with college level or higher education, percentage of residents 65 years and older, percentage of residents who were agriculture workers, and the number of physicians per 100,000 people (15). The residential areas of patients were categorized as urban, suburban, and rural in this survey. Baseline comorbidities were determined by individual medical records in the year preceding cohort entry, all of which were assessed by the established Charlson–Deyo comorbidity index (CCI) (16). The CCI score consisted of 17 chronic diseases, each with a score of 1–6 points. The sum of these scores is regarded as a measure of the burden of comorbidities, with higher scores indicating more severe impact of the comorbidities. To avoid double counting and possible overadjustment in the regression model, RA was excluded from the CCI score.

### Statistical Modeling

Data were analyzed using SAS software Version 9.3 (SAS Institute Inc, Cary, NC, USA). Descriptive and inferential statistical analyses were performed in accordance with the study aims and the nature of variables. For descriptive analysis, the distributions of demographic data and disease characteristics between the acupuncture users and non-users were evaluated using the Chi-square test and Student's *t*-test, as appropriate. The incidence of endometriosis per 1,000 person-years during the follow-up period was calculated. The Kaplan–Meier method was employed to plot the cumulative incidence of endometriosis during the follow-up period, and the log-rank test was used to assess the difference between the groups. For inferential analysis, Cox proportional hazards regression analysis was applied to compute the hazard ratio (HR) with 95% confidence interval (CI) of endometriosis risk in association with acupuncture use. To further assess the robustness of the association of acupuncture use with subsequent endometriosis risk, we performed a subgroup analysis, dividing acupuncture use into low intensity (1–3 packages), medium intensity (4–14 packages), and high intensity (≥15 packages), to explore if acupuncture exerts a dose-response effect against endometriosis. Assumptions of the proportional hazards model were verified using plots of log (–log [survival]) vs. log (time) and Schoenfeld residuals vs. time. Two-tailed *p* < 0.05 was considered significant.

## Results

The acupuncture user and non-user cohorts each provided data for 2,407 subjects. The mean age of the enrollees was 53.80 ± 13.37 years (Table 1). The majority of participants had a monthly income of NTD 17,881–NTD 43,900 (51.7%) and lived in urbanized areas (57.2%). Regarding comorbidities, the mean CCI score was 4.58 (± 3.61). No significant differences were found between the two groups with respect to age, monthly income, location of residence, or CCI score after propensity score matching, indicating that the two groups were comparable in baseline characteristics.

**Table 1:** Subject demographic data and comorbidities.

	**Total subjects**	**AP non-users**	**AP users**	***P***
		***n* = 2407 (%)**	***n* = 2407 (%)**	
Age (year)				0.76
Mean (SD)	53.80 ± 13.37	53.86 ± 13.40	53.74 ± 13.38	
Age				0.50
≤ 50 years	1919 (39.9)	948 (39.4)	971 (40.3)	
>50 years	2895 (60.1)	1459 (60.6)	1436 (59.7)	
Monthly income				0.80
Low	2206 (45.8)	1102 (45.8)	1104 (45.9)	
Median	2487 (51.7)	1248 (51.8)	1239 (51.5)	
High	121 (2.5)	57 (2.4)	64 (2.7)	
Residential area				0.49
Urban	2752 (57.2)	1415 (58.8)	1455 (60.4)	
Suburban	757 (15.7)	389 (16.2)	368 (15.3)	
Rural	1305 (27.1)	603 (25.1)	584 (24.3)	
CCI				0.21
Mean (SD)	4.58 (3.61)	4.73 (3.99)	4.42 (3.22)	

*AP, acupuncture; SD, standard deviation*.

**Figure 2:**
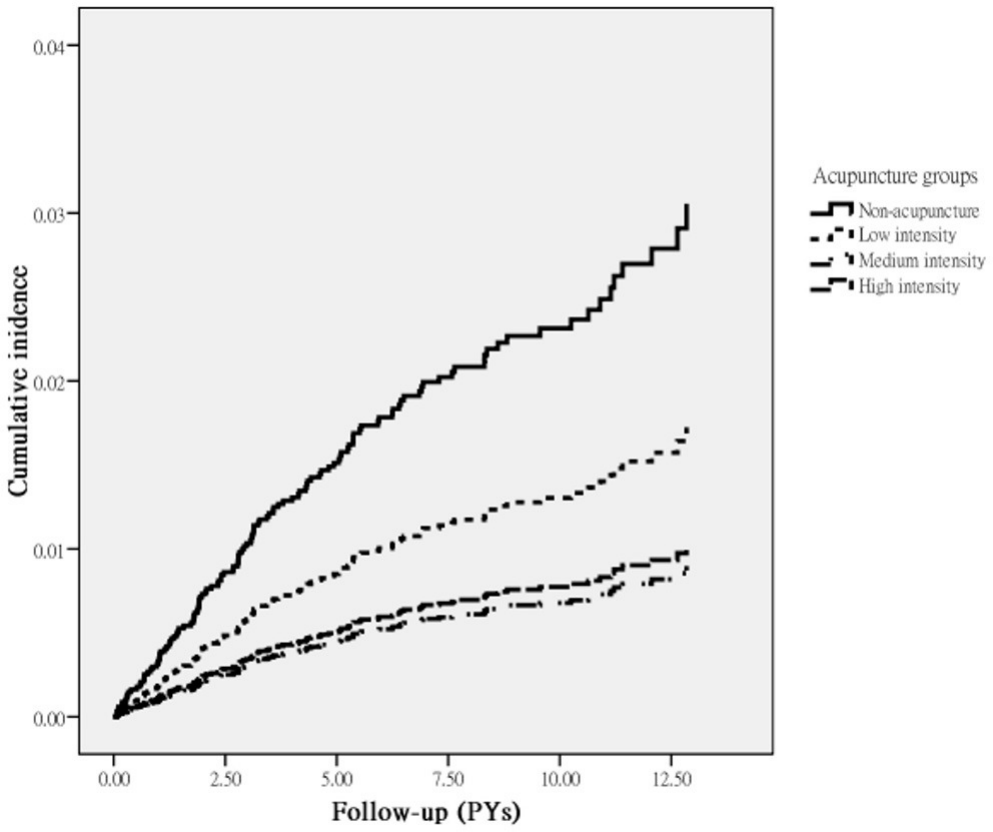
Incidence of endometriosis in RA patients according to acupuncture use.

Review of the full cohort identified 129 episodes of endometriosis, with 94 occurring in acupuncture non-users and 35 in acupuncture users during follow-up periods of 19,139.66 and 14,772.27 person-years, respectively. The incidence of endometriosis was significantly lower in the acupuncture users than in non-users (2.36 vs. 4.91, respectively, per 1,000 PYs) (adjusted HR = 0.45; 95% CI: 0.31–0.65) (Table 2). Notably, subgroups with low, medium, and high intensity of acupuncture use were associated with a lower risk of endometriosis, and a dose-dependent relationship was identified between acupuncture intensity and the risk of endometriosis (Table 2). Results of Kaplan–Meier survival analysis and the log-rank test also showed a statistically significant difference in the survival rate (free of endometriosis) between the three groups and non-users during the follow-up period. Those receiving acupuncture of higher intensity had a significantly lower incidence rate of endometriosis than did those who did not receive acupuncture (*p* < 0.001) (Figure 2).

**Table 2:** Risk of endometriosis according to intensity of acupuncture use.

**AP intensity**	**Number**	**Event**	**PYs**	Incidence^*^	**Crude HR (95% CI)**	Adjusted HR^**^ (95% CI)
Without AP	2407	94	19139.66	4.91	1	1
With AP	2407	35	14772.27	2.36	0.46 (0.31-0.68)	0.45 (0.30–0.65)
Low intensity	1391	23	7476.25	3.08	0.57 (0.37–0.90)	0.56 (0.35–0.88)
Medium intensity	551	6	3623.55	1.66	0.34 (0.15–0.72)	0.33 (0.14–0.72)
High intensity	465	6	3672.46	1.63	0.32 (0.14–0.71)	0.29 (0.13–0.66)
*p* (Schoenfeld test)						0.85

*AP, acupuncture; HR, hazard ratio; CI, confidence interval*.

* *Incidence per 1,000 person-years (PYs)*.

** *Model adjusted for age, residential area, monthly income, medication use, and Charlson-Deyo comorbidity index*.

## Discussion

The findings of this retrospective 10-year follow-up study indicated that the risk of endometriosis in RA patients was lower for those who received acupuncture than for those who did not. Multivariable analysis showed that acupuncture use was associated with a 55% lower risk of endometriosis in women with RA. High-intensity acupuncture had the greatest benefits, with a 71% lower risk of endometriosis. As the demonstration of a dose-response relationship is considered strong evidence for a causal relationship between the exposure level and the outcome, this finding suggests that acupuncture use is successful in lowering the risk of endometriosis. While studies on this relationship are scarce, this positive therapeutic effect is consistent with earlier reports and further adds to a growing body of literature regarding the clinical efficacy of acupuncture (11, 17, 18).

Several studies have addressed the premise that changes in the immune system play a crucial role in the comorbid relationship between endometriosis and autoimmune diseases (6, 7, 19). Women affected by endometriosis are more likely than those who were not affected by it to have higher levels of proinflammatory cytokines such as interleukin-1 beta (IL-1), interleukin-6 (IL-6), and tumor necrosis factor-α (TNF-α) occurring locally, in the peritoneal cavity, and systemically (20, 21). Nowadays, acupuncture is gaining acceptance among RA individuals (10, 22), and among the reasons why acupuncture is adopted by medical professionals include its beneficial anti-inflammatory effect and its regulation of immune system function (11, 12).

Based on findings yielded by studies conducted thus far, we propose that the mechanisms by which acupuncture exerts marked anti-inflammatory effects may be related to the inhibition of nuclear factor kappa beta (NF-κB) and mitogen-associated kinase (MAPK) signaling pathways. Studies in murine models found that acupuncture suppressed the production of IL-6 and TNF-α in plasma by inhibiting the activation of NF-κB (23, 24). It is well known that activated NF-κB binds specific κB sequences in the promoter region of inflammatory factor genes, inducing transcription of TNF-α, IL-1, and IL-6 (25). These inflammatory factors induce the proliferation of endometriotic cells, thus contributing to the development of endometriosis (5, 7). Alternatively, several review articles have concluded that the association between acupuncture and lower inflammatory parameters occurs through the MAPK signaling pathway (26, 27). This pathway is involved in a diverse array of cellular processes that include inflammation, angiogenesis, and proliferation (28). Using a rodent model, Fang and colleagues found that acupuncture markedly reduced pain due to inflammatory responses via down-regulation of p38 MAPK (29). Consequently, MAPK family proteins have been viewed as a new class of therapeutic targets for the prevention and treatment of endometriosis (7).

While our study is the first to investigate the relationship between acupuncture use and the subsequent risk of endometriosis in women with RA, several noteworthy limitations should be considered. First, our results were obtained using ICD-9-CM diagnostic codes in patient medical records. Thus, a number of relevant cases may have been misclassified. To minimize this bias, we enrolled only patients with new-onset RA or endometriosis and only after the patients had at least 3 outpatient visits that reported consistent diagnoses or at least 1 inpatient admission. In addition, the NHI of Taiwan randomly reviews charts and audits medical charges to verify the accuracy of claims (13). Further, the two study groups were identical in the coding approach and data availability; therefore, any misclassification bias is likely to have been non-differential to possibly underestimate rather than overestimate the observed differences. Second, no reliable index of RA severity is available from LHID, and failure to adjust for this factor may bias the findings. To address this concern, two sensitivity analyses were performed to robustly identify the relationship between acupuncture use and the subsequent risk of endometriosis. The first sensitivity analysis was limited to female patients with no comorbidities. The analysis indicated that acupuncture still protected against development of endometriosis (adjusted HR=0.49; 95% CI: 0.32–0.76). In the second analysis, we used the prescription of biological agents as an indicator of RA severity in the sensitivity analysis and separated all enrollees based on whether they received biological agents for more, or less, than 6 months following RA diagnosis. Biological agents were used by 43.8% of the acupuncture users (1054/2407) and 44.7% (1076/2407) of the non-users. Analysis, taking into consideration the use of biological agents, yielded findings that were essentially the same as those reported in the original analysis (adjusted HR = 0.47; 95% CI: 0.32–0.69). Third, the LHID lacks information on such variables as social network relationships, family history, laboratory data, and educational level. Thus, future studies that control for these variables are worthy of further pursuit. Fourth, a surveillance bias might occur in this survey, as the acupuncture users may have had a greater chance of being diagnosed with endometriosis than their control counterparts. To address this issue, we calculated the frequency of medical visits for each study subject and adjusted this variable in the multivariate regression model. The results of the reanalysis revealed that the positive effects of acupuncture slightly decreased but were still statistically significant, with an adjusted HR of 0.54 (95% CI: 0.36–0.80), suggesting that the number of ambulatory care visits did not appreciably affect the relationships reported earlier. Fifth, although our study revealed a substantial beneficial effect of acupuncture on decreasing endometriosis risk among RA patients, it should be noted that participants were not initially randomly categorized into users and nonusers and were recruited from a single country only. Therefore, caution should be used when interpreting the findings. Randomized controlled trials using patients from additional countries are recommended to corroborate the present findings and to elucidate the mechanisms underlying the beneficial effects of acupuncture.

These limitations notwithstanding, this study has several strengths. First, the available data were obtained from the Taiwanese NHI database, which is a government-run, single-payer HNI program covering more than 99% of insured persons and healthcare institutes throughout Taiwan, ensuring that the study cohort is representative of the general population with minimal selection bias. Second, the large number of subjects, together with a strict protocol for disease assessment, allowed us to achieve adequate statistical power for psychometrically sound analysis, especially given the relatively low incidence of RA in the population.

## Conclusion

The findings from real-world data revealed that women with RA who receive acupuncture have a lower likelihood of developing endometriosis than those without acupuncture use. Future prospective randomized trials, which directly address the limitations of this study, are needed to provide more conclusive evidence of the findings reported in this study, thus paving the way for further studies regarding the effects of acupuncture on patients with other chronic medical conditions.

## Data Availability Statement

The raw data supporting the conclusions of this article will be made available by the authors, without undue reservation.

## Ethics Statement

The studies involving human participants were reviewed and approved by Institutional Review Board and Ethics Committee of Buddhist Dalin Tzu Chi Hospital, Taiwan (No. B10004021-3). Written informed consent for participation was not required for this study in accordance with the national legislation and the institutional requirements.

## Author Contributions

W-JC, C-HH, HL, N-SL, and T-YT: study concept and design. N-SL and T-YT: acquisition of data. HL, T-YT, and H-RG: data analysis. N-SL, Y-TH, and T-YT: project management. W-JC, HL, H-RG, T-YT, C-HH, and N-SL: writing. All authors contributed to the article and approved the submitted version.

## Conflict of Interest

The authors declare that the research was conducted in the absence of any commercial or financial relationships that could be construed as a potential conflict of interest.
